# Comparison between nafamostat mesilate and unfractionated heparin as anticoagulant during continuous renal replacement therapy

**DOI:** 10.1186/cc14384

**Published:** 2015-03-16

**Authors:** S Makino, H Kita, Y Miyatake, T Yokoyama, K Kubota, N Obata, M Egi, T Misumi, S Izuta, S Mizobuchi

**Affiliations:** 1 Kobe University Hospital, Kobe, Japan

## Introduction

For continuous renal replacement therapy (CRRT), continuous administration of anticoagulant would be necessary to prevent the circuit clotting. Nafamostat mesilate (NM) is commonly used as its anticoagulant in Japan, although unfractionated heparin (UFH) is the most frequently used anticoagulant internationally. There is little study to compare the risk and benefit of NM with UFH as an anticoagulant during CRRT.

## Methods

We conducted a single-center retrospective observational study to compare NM with UFH as anticoagulant during CRRT. We screened subsequent critically ill patients requiring CRRT in our ICU from January 2011 to December 2013. We excluded patients who required any other extracorporeal circuit including extracorporeal membrane oxygenation, who used both NM and UFH simultaneously, or who were administered any other anticoagulant including gabexate mesilate or urokinase. The primary outcome of this analysis was filter life, and the secondary outcome was the incidence of bleeding complications during CRRT. As an initial dose, NM and UFH were given pre filter at 15 to 25 mg/hour and 1,500 to 3,000 IU/hour, respectively. The dose of both drugs was adjusted to maintain activated clotting time at post filter between 150 and 200 seconds. Filter life was assessed using the Kaplan-Meier method and the incident of bleeding complications was compared using the chi-square test. *P *< 0.05 was considered to be statically significant.

## Results

We included 101 patients in this study. Among them, 76 patients were with NM and 25 patients were with UFH. They used 239 filters in total; 173 with NM, 66 with UFH. There were significantly more postsurgical patients in the NM group (*P *= 0.001). There was no difference in age, APACHE II score, days from ICU admission to commencement of CRRT, length of ICU stay and mortality between two groups. There was no difference in median number of filters used by one patient (NM vs. heparin; median of 1.5 (IQR) vs. 2 (IQR), *P *= 0.27). Filter life in the UFH group was significantly longer than those in the NM group (NM vs. UFH; median of 24 hours vs. 36 hours; *P *= 0.01). The incidence of bleeding complications was not significantly different between two groups (*P *= 0.15).

## Conclusion

In our retrospective analysis with 101 patients, filter life with UFH was significantly longer than those with NM. The incidence of bleeding complications was not significantly differed between patients with NM and UFH.

